# Genetic Analysis and Molecular Breeding Applications of Malting Quality QTLs in Barley

**DOI:** 10.3389/fgene.2019.00352

**Published:** 2019-04-24

**Authors:** Yunxia Fang, Xiaoqin Zhang, Dawei Xue

**Affiliations:** College of Life and Environmental Sciences, Hangzhou Normal University, Hangzhou, China

**Keywords:** malting quality, quantitative trait loci, phenotypic variance, correlation, marker-assisted selection, barley

## Abstract

Malting quality is an important determinant of the value of barley grain used in malting and brewing. With recent sequencing and assembling of the barley genome, an increasing number of quantitative trait loci (QTLs) and genes related to malting quality have been identified and cloned, which lays a good molecular genetic basis for barley quality improvement. In this review, we describe the following indicators of malting quality: malt extract (ME), diastatic power (DP), kolbach index (KI), wort viscosity (VIS), free amino nitrogen (FAN) content, soluble protein (SP) content, wort β-glucan (WBG) content, and protein content (PC), and have list related QTLs/genes with high phenotypic variation in multiple populations or environments. Meanwhile, the correlations among the quality parameters and parts of significant indicators suitable for improvement are discussed based on nutrient composition and content required for high-quality malt, which will provide reference for molecular marker-assisted selection (MAS) of malting quality in barley.

## Introduction

Barley (*Hordeum vulgare*) is the fourth largest cereal crop in the world, widely used for livestock feed, food, and industrial utilization (Bond et al., 2015; FAOSTAT^1^). In industrial applications, barley is processed into malt and mainly used for brewing and distilling, in which malting quality is an important factor in determining the quality of the manufactured products (Kochevenko et al., 2018). About 30% of the barley produced globally is used for malting, thus breeding barley varieties with high-quality malt for processing is an important goal (Bond et al., 2015; Walker and Panozzo, 2016; Kochevenko et al., 2018).

In barley endosperm, the nutrients such as starch and protein are stored and directly determine the barely quality (Bamforth, 2003; Jamar et al., 2011). During seed germination, the gibberellic acid (GA), released by the embryo, induces a large number of hydrolases in aleurone layer and begin to degrade the endosperm cell wall (Zentella et al., 2002; Bamforth, 2017). Then, many hydrolases enter into the endosperm cells and start to degrade proteins, starches, and lipids. In the procession, the conversion of nutrients has always continued, and low molecular weight sugars, amino acids, fatty acids, and enzymes are formed, which provide substances for subsequent fermentation. Among them, the amount and quality of the converted substances determine the malting quality (Autio et al., 2001; Georg-Kraemer et al., 2001; Bamforth, 2009).

In order to provide a genetic basis for the breeding of high-quality barley varieties, we focus on the known major quantitative trait loci (QTLs) related to malting quality and systematically analyze the association between the quality traits and the improvement prospects in this paper.

## Composition of Malting Quality Traits in Barley

Malting quality is mainly determined by malt extract (ME), diastatic power (DP), viscosity (VIS), wort β-glucan (WBG) content, kolbach index (KI), free amino nitrogen (FAN) content, soluble protein (SP) content, and grain protein (GP) (Cu et al., 2016). ME, the ratio of extracted malt soluble matter to dry malt weight, is directly related to malt production, with a higher ME being required for better malting quality (Sarkar et al., 2008). DP represents the ability to hydrolyze starch to simple sugars during barley germination, which is directly proportional to the yield and quality of beer brewing (Henson and Duke, 2007). KI, also known as malt protein solubility, is an indispensable parameter of malting quality. Viscosity reflects the solubility and filtration speed of the malt wort, and low VIS value is an important indicator of high-quality malt. β-Glucan is the main component of the endosperm cell wall, and high concentration of WBG will hinder the hydrolysis process in malt. FAN content contains amino acids and small peptides from protein degradation by protease, and is the only nitrogen source for yeast growth. In addition, the protein content (PC) in the grain is also a significant factor influencing malting quality. All of these traits in combination affect the malting quality.

Besides the genetic factors, malting quality is also affected by environmental conditions (Qi et al., 2005; Chen et al., 2006), and there are complex inhibitory relationships among malting quality traits. For instance, the GP in the grains is negatively correlated with ME and positively correlated with DP (Eagles et al., 1995; Mather et al., 1997). Therefore, it is difficult to directly locate and clone the malt quality-related genes. However, as an effective strategy, QTL analysis had been widely used in identification and localization of QTLs in different crops, but large genome size and high homology limited polymorphic DNA markers development for the establishment of the genetic map in barley. Excitingly, with the completion of the assembly of the barley genome, many QTLs regulating malting quality traits have been located in smaller intervals using various genetic markers, and some essential genes have been cloned. Up to now, More than 200 malting quality QTLs/genes have been reported (Wei et al., 2009), but only a small number of QTLs/genes have been successfully applied in molecular breeding (Han et al., 1997; Igartua et al., 2000; Rae et al., 2007; Li et al., 2010; Xu et al., 2018). One reason may be the population size, thus the linkage of unfavorable genetic traits will reduce the accuracy of QTL screening in small populations (Zhang et al., 2012; Cu et al., 2016). On the other hand, the QTLs accounting for lower phenotypic variation are easily affected by environmental factors, which are inconvenient for breeding selection.

## Major Qtls for Malting Quality Traits

### Malt Extract (ME)

Malt extract includes the soluble matters produced by malt itself and the enzymatic hydrolysis during saccharification and fermentation, which reflects the degree of malt dissolution and the amount of enzyme formation in the malting process. ME is quantitative trait controlled by multiple genes, which also vary among different varieties. At present, a number of QTLs related to ME with high variances have been identified and located on chromosomes 1, 2, 4, 5, and 7 (Table 1).

**Table 1:** QTLs/Genes identified for malting quality in barley.

		Location	Flanking		Phenotypic
Traits	Chromosome	(cM)	markers	Populations	variation	QTL/gene	References
ME	1H	38	bPb-9423	Triumph × Morex^∗^	15.9–31.0%	N.A.	Elía et al., 2010
	1H	60.3	Bmag0211	Nure × Tremois	21.1%	*Qme1.1*	Laidò et al., 2009
	1H	89.9	cor18	Nure × Tremois	11.3%	*Qme1.2*	Laidò et al., 2009
	2H	29	GBM1121	TX9425 × Naso Nijo^∗^	48.4%	*QMe.NaTx-2H/MLOC_60943.2*	Wang J. et al., 2015
	2H	88.55	vrs1	Triumph × Morex^∗^	34.8%	N.A.	Elía et al., 2010
	3H	66	bPb-8480	Triumph × Morex^∗^	19.2%	N.A.	Elía et al., 2010
	4H	30	BCD402B	Steptoe × Morex	37.6%	QTL2 (*HvTLP8*)	Gao et al., 2004; Singh et al., 2017
	4H	45–54	2_1122–1_0411	Vlamingh^∗^ × Buloke	7.9–13%	N.A.	Walker et al., 2013
	4H	62-64	1_1244–2_0361	Vlamingh^∗^ × Buloke	3.5–10.3%	N.A.	Walker et al., 2013
	5H	14	HvHEMH1	Triumph^∗^ × Morex	12.5–14.4	N.A.	Elía et al., 2010
	5H	141–150	GA20-2978	Mikamo golden × Harrington^∗^	35.7–53.6%	N.A.	Zhou et al., 2012
	7H	122	bPb-3484	Triumph × Morex^∗^	14.1–21.7%	N.A.	Elía et al., 2010
DP	1H	4.7	12_31144	MSU and CAP panels	1.57%	α-Glucosidase (*Aglu3*)	Pauli et al., 2015
	2H	159.6	12_10487	MSU and CAP panels	N.A.	α-Glucosidase (*Aglu5*)	Pauli et al., 2015
	3H	13.4	12_30818	N.A.	N.A.	α-Glucosidase (*Aglu2*)	Szűcs et al., 2009
	7H	90.6	GBM1419	N.A.	N.A.	α-Glucosidase (*Agl97*)	Stanley et al., 2011; Andriotis et al., 2016
	1H	50.56	009-148	Admiral × Navigator^∗^	14.31–15.52%	α-Amylase	Cu et al., 2016
	2H	122	BMAG125	Amazone × St. 2730e × Kym	11.2%	*QAa.S42-2H.a*	von Korff et al., 2008
	4H	14	HVM40	Amazone × St. 2730e × Kym	13.2%	*QAa.S42-4H.a*	von Korff et al., 2008
	4H	190	HDAMYB	Amazone × St. 2730e × Kym	15.8%	*QAa.S42-4H.c*	von Korff et al., 2008
	5H	119.4	Xp11m48B327	Baudin × AC Metcalfe^∗^	25.6%	α-Amylase	Zhou et al., 2016
	5H	187.52	12_10322^i^	Two-row spring breeding lines	12.4%	α-Amylase	Mohammadi et al., 2015
	6H	89.1	Amy1	Harrington × TR306	N.A.	α-Amylase 1	GrainGenes3.0
	7H	122	bPb-3484	Triumph × Morex^∗^	16.0%	α-Amylase 2	Elía et al., 2010
	2H	64.68	Bmy2	Steptoe × Morex	N.A.	β-Amylase 2	Han et al., 1995
	3H	140.3–142.8	bPb-4564-bPb-3634	CM72 × Gairdner	12.81%	β-Amylase, *qBAM3*	Wei et al., 2009
	4H	74.2	TP2729	Admiral^∗^ × Navigator	11.89%	β-Amylase	Cu et al., 2016
	4H	134.64	bPb-9820	Admiral^∗^ × Navigator	30.74–49.66%	β-Amylase 1	Cu et al., 2016
	5H	78.4	GBM1039	TX9425 × Naso Nijo^∗^	20.9%	*QDp.NaTx-5H*	Wang J. et al., 2015
	7H	23.10	HVWAXYG	TX9425 × Naso Nijo^∗^	13%	*QDp.NaTx-7H*	Wang J. et al., 2015
	7H	60.9	HvSS1		N.A.	Sucrose synthase 1 (*SS1*)	Szűcs et al., 2009; GrainGenes 3.0
	5H	174–178.4	bPb-4809-bPb-5766	CM72^∗^ × Gairdner	19.4%	Limit dextrinase, *qLD5*	Wei et al., 2009
	7H	58.7 c	bPb-2866	Yerong × Franklin	31.0%	Limit dextrinase	Wang J. et al., 2015; Wang X. et al., 2015
KI	1H	50.56	009-148	Admira l × Navigator^∗^	9.47%	N.A.	Cu et al., 2016
	2H	82	vrs1	Triumph × Morex^∗^	16.7–24.1%	N.A.	Elía et al., 2010
	3H	57.11	SCRI_RS_115045	Victoriana × Sofiara	13.74%	*QKOL-3*	Kochevenko et al., 2018
	4H	15.76–23.98	TP4209-HvPhBBA	Admiral^∗^ × Navigator	6.52–11.5%	N.A.	Cu et al., 2016
	5H	141–150	GA20-2978	Mikamo golden × Harrington^∗^	56.8–77.5%	N.A.	Zhou et al., 2012
	6H	57.20	2259-488	Stellar × 01Ab8219^∗^	23.40%	N.A.	Islamovic et al., 2014
	7H	10.7	bPb-4725	TX9425 × Naso Nijo^∗^	15.4%	*QKi.NaTx-7H*	Wang J. et al., 2015
VIS	1H	60.3	Bmag0211	Nure × Tremois	39.5%	*Qvis1.1*	Laidò et al., 2009
	3H	63.66	BK_08	Victoriana × Sofiara	13.82%	QVIS-3	Kochevenko et al., 2018
	4H	0.00	12_30540	Victoriana × Sofiara	8.48%	N.A.	Kochevenko et al., 2018
	5H	137.5	E38M50-215	Nure × Tremois	24.3%	*Qvis5.1*	Laidò et al., 2009
	7H	20.79	bPb-5902	TX9425^∗^ × Naso Nijo	14.5%	*QVi.NaTx-7H*	Wang J. et al., 2015
	7H	68.03–72.14	TP1236-TP11077	Admiral × Navigator^∗^	6.31–15.05%	N.A.	Cu et al., 2016
	7H	125.4	bPb-1669	174 European barley cultivars	N.A.	N.A.	Matthies et al., 2014
WBG	1H	63.5	Bmag035	Arapiles × Franklin	36.0–52.8%	N.A.	Panozzo et al., 2007
	1H	58.06	11_10176	Six-row spring breeding lines	32.5%	N.A.	Mohammadi et al., 2015
	2H	65.6	Ebmac0684	Alexis × Sloop	11.4–35.2%	N.A.	Panozzo et al., 2007
	2H	58.0–69.4	Adh8-Bmy2	Steptoe × Morex	19.2%	*CslF*	Han et al., 1995; Burton et al., 2006
	3H	25	N.A.	Harrington × TR306	12%	N.A.	Mather et al., 1997
	5H	187	11_20402	Two-row spring breeding lines	36%	N.A.	Mohammadi et al., 2015
	6H	35–55	1969–4070	Mikamo golden^∗^ × Harrington	13.8–17.2%	N.A.	Zhou et al., 2012
	7H	52.3	HVM4	Yonezawa Mochi × Neulssalbori	44.4%	N.A.	Kim et al., 2011
	7H	71.0	opU01	Yonezawa Mochi × Neulssalbori	31.6–37.6%	N.A.	Kim et al., 2011
	7H	91.3	CDO673	‘Bowman’ × OUM125	N.A.	*HvCslF6*	Taketa et al., 2011
	1H	50.9	Bmag0211	Arapiles × Franklin	14.6–33.8%	β-Glucanase (*HvBDG*)	Panozzo et al., 2007; Pauli et al., 2015
	1H	61.2–77.6	ABG494-ABC160	Steptoe × Morex.	12.3%	β-Glucanase (*Glb 1*)	Han et al., 1995; GrainGenes 3.0
	4H	22.9	GMS89	Alexis × Sloop	12.1–40.3%	β-Glucanase	Panozzo et al., 2007
	4H	79.5	ABG484-WG464	Steptoe × Morex.	12.6%	β-Glucanase	Han et al., 1995
	7H	73.1	Ale-ABC302	Steptoe × Morex.	10.8%	β-Glucanase (*Glb 2*)	Han et al., 1995; Jamar et al., 2011
FAN	1H	50.56	009-148	Admiral × Navigator^∗^	11.7%	N.A.	Cu et al., 2016
	1H	50.9	Bmag0211	Arapiles × Franklin	64.0%	N.A.	Panozzo et al., 2007
	1H	57.8	Bmag0345	Arapiles × Franklin	39–60%	N.A.	Panozzo et al., 2007
	3H	85	P14.M61.154	Alexis × Sloop	11.1–19.7%	N.A.	Panozzo et al., 2007
	5H	118.8	GMS002	Baudin × AC Metcalfe^∗^	31.9%	N.A.	Zhou et al., 2016
	5H	185	P11.M51-193	Alexis × Sloop	4.9–20.6%	N.A.	Panozzo et al., 2007
	7H	20.79–43.47	HVWAXYG	TX9425 × Naso Nijo^∗^	11.5%	*QAn.NaTx-7H*	Wang J. et al., 2015
	7H	34	bPb-7183	Stellar^∗^ × 01Ab8219	16.71%	N.A.	Islamovic et al., 2014
	7H	66.16–73.26	TP10224-TP7945	Admiral^∗^ × Navigator	13.90–14.95	N.A.	Cu et al., 2016
SP	1H	50.56	009-148	Admiral × Navigator^∗^	11.66–12.15	KI, FAN and SP	Cu et al., 2016
	1H	94.9	bPb-6911	174 European barley cultivars	N.A.	N.A.	Matthies et al., 2014
	3H	60.96	BK_08	Victoriana × Sofiara	27.31%	QSNI-3-1	Kochevenko et al., 2018
	5H	117.9	GMS001	Baudin × AC Metcalfe^∗^	33.6%	N.A.	Zhou et al., 2016
	5H	184.4	bPb-1217	174 European barley cultivars	N.A.	N.A.	Matthies et al., 2014
	7H	62.8–66.16 c	TP3252-TP1819	Admiral^∗^ × Navigator	12.41–14.37%	N.A.	Cu et al., 2016
	7H	236	N.A.	Harrington × TR306	29%	N.A.	Mather et al., 1997
MPC	2H	43.27	GBMS229	Brenda × HS213^∗^	14.72%	*Qpc2.1*	Li et al., 2005
	7H	106.6	Bmag120	Brenda × HS213^∗^	12.02%	*Qpc7.1*	Li et al., 2005
GPC	1H	108.7	Bmag0382	Nure × Tremois	29.1%	*Qpc1.1*	Laidò et al., 2009
	2H	82	vrs1	Triumph^∗^ × Morex	44.5–62.3%	N.A.	Elía et al., 2010
	3H	109.2	bPb-3630	Triumph × Morex^∗^	15.8–17.2%	N.A.	Elía et al., 2010
	6H	117.9	OPA17b	Nure × Tremois	18.7%	*Qpc6.1*	Laidò et al., 2009
	7H	55	E37M47_g	Triumph^∗^ × Morex	13.4–16.2%	N.A.	Elía et al., 2010

*The rows indicate the traits name, chromosome, located region, the closest markers or marker interval, population source, phenotypic variance, QTLs/genes name and references. ^∗^Indicates the source of increasing allele. N.A. indicates related informations were not founded*.

Using a double haploid (DH) population, Wang J. et al. (2015) identified two QTLs, in which the *QMe.NaTx-2H* was mapped in the 24–35 cM region of chromosome 2 (2H) and explained 48.4% of the total phenotypic variance. A cell wall hydrolytic enzyme, endo-1,4-xylanase A (MLOC_60943.2), was found near the marker GBM1121, which was closely linked to *QMe.NaTx-2H*. The main function of this gene is to degrade the endosperm cell wall and facilitate other substances more easily digestible in the cells, so it is suspected to be the target gene regulating ME. Another QTL, detected on 1H, contributed little to phenotypic variance and was not identified in other environments. Laidò et al. (2009) detected a QTL *Qme1.1* on 1H, which was positioned at 60.3 cM and explained 21.1% of the phenotypic variance. Using different population, a ME-associate QTL, with extremely high phenotypic variance, was also detected in this region, which verifies that there is a major QTL for ME in the interval (Panozzo et al., 2007; Laidò et al., 2009). Matthies et al. (2014) analyzed 174 European barley cultivars by genome-wide association analysis (GWAS) and also identified a major QTL near *Qme1.1*. They found that this QTL was not only related to ME but also regulated VIS. Elía et al. (2010) and Wang J. et al. (2015) mapped two QTLs on short and long arm of 2H, respectively, both of which exhibited high phenotypic variances. Singh et al. (2017) cloned a major QTL *thaumatin-like protein 8* (*TLP8*) near the telomeric region of 4H, which acts on β-glucan through redox reaction, thereby affecting the ME. In addition, two closely spaced QTLs were also detected on 4H, explaining approximately 8–13% and 4–10% of the variances, respectively (Walker et al., 2013). On 5H, two QTLs for ME were identified, and one accounted for 35.7–53.6% of the variance (Elía et al., 2010; Zhou et al., 2012).

### Diastatic Power (DP)

Diastatic power is a critical parameter of malting quality. In general, a higher DP is required for better malting quality and a higher ME (Henson and Duke, 2007). The DP of barley malt represents the collective activity of four starch-degrading enzymes, namely α-amylase, β-amylase, limit dextrinase, and α-glucosidase (Gibson et al., 1995; Walker and Panozzo, 2016). The conversion of starch to fermentable products in the endosperm is primarily catalyzed by α-amylase, followed by β-amylase, limit dextrinase, and α-glucosidase (Bamforth, 2009). During this process, DP is significantly positively correlated with amylase activity, which can be determined by measuring the activity of amylase. Therefore, the identification of QTLs/genes related to amylase activity and its application in breeding is one effective means of improving malting quality.

Diastatic power, not highly influenced by environmental conditions, is mainly determined by genetic factors and easier to improve. In recent years, multiple major DP-related QTLs have been mapped or cloned using positional cloning and comparative genomics method (Table 1). Eight major QTLs influencing α-amylase activity have been listed, and two of which, encoding α-amylase1 and α-amylase2, have been cloned (Elía et al., 2010; GrainGenes 3.0). By SNP-based maps, von Korff et al. (2008) identified three QTLs and each accounted for about 10% of phenotypic variances. Two major QTLs for α-amylase were located on 5H, and explained 25.6% and 12.4% of the phenotypic variances, respectively (Mohammadi et al., 2015; Zhou et al., 2016). Cu et al. (2016) mapped three QTLs that significantly increased DP on 1H and 4H. The QTL, located on 1H at 50.56 cM, accounted for 15% of the variance and could critically increase α-amylase activity. Another two QTLs, which significantly increased the β-amylase activity, were identified on 4H and contributed to 11.89% and 30.74–49.66% of the phenotypic variances, respectively. The QTL linked to bPb-9820, which encodes β-amylase 1, has been cloned and can be used for improving malting quality (Cu et al., 2016). Another cloned β-amylase gene is *β-amylase 2*, which was located at 64.68 cM on 2H (Elía et al., 2010). QTL *qBAM3*, mapped on 3H between markers bPb-4564 and bPb-3634, was also linked to β-amylase activity and explained 12.81% of the phenotypic variance (Wei et al., 2009).

α-Glucosidase is an essential enzyme in the starch degradation pathway, and four α-glucosidase genes have been cloned. A gene immediately related to DP was found at 4.7 cM on 1H by GWAS. *Aglu3*, encoding an α-glucosidase, was found in this interval and contributes to the conversion of gelatinized starch and glucan to sugars; however, the genetic effect of *Aglu3* is only 1.57% (Pauli et al., 2015). *HvAGL197*,α-glucosidase-related gene, is involved in the conversion of maltose to glucose instead of starch degradation (Stanley et al., 2011). *Aglu2* and *Aglu5*, another two α-glucosidase-related genes, were located on 3H and 2H (Szűcs et al., 2009; Pauli et al., 2015). Two major QTLs controlling limit dextrinase activity were positioned on 5H and 7H, respectively. The QTL *qLD5* was located on 5H at 174–178.4 cM, explaining 19.4% of the phenotypic variance (Wei et al., 2009). The last QTL was positioned at 58.7 cM on 7H and accounted for 31.0% of the phenotypic variance, which is significantly higher compared to the other two QTLs (Wang X. et al., 2015).

### Kolbach Index (KI)

Kolbach index is typically measured as the ratio of soluble nitrogen to total nitrogen in the wort. In brewing applications, the degree of protein degradation in barley malt will have distinct effects on yeast growth and wort filtration. When the degree of protein degradation is low, the corresponding enzyme activity is also reduced, resulting in lower ME, protein turbidity, and wort filtration difficulty. When decomposition is high, the corresponding KI is also increased and the normal proportion of protein components is compromised, resulting in accelerated yeast aging and thin beer taste. Therefore, the KI of elite malt should be controlled between 41 and 48% (Molina-Cano et al., 1997).

Although KI is affected by environmental conditions, the genetic background of different varieties is also significant. Using genetic populations, QTLs contributing to high variances were identified on 1–7H under multiple environmental conditions (Elía et al., 2010; Zhou et al., 2012; Islamovic et al., 2014; Wang J. et al., 2015; Kochevenko et al., 2018) (Table 1). Among them, the QTL on 5H was positioned at an interval of 141–150 cM and accounted for 56.8–77.5% of the variance, which is much higher than that on other chromosomes (Wang J. et al., 2015). Secondly, QTL, on 6H at 57.20 cM, explained 23.40% of the phenotypic variance (Islamovic et al., 2014). These two major QTLs have great potential in breeding applications. However, no KI-related genes have thus far been cloned.

### Viscosity (VIS)

The main components of the cell wall of barley endosperm are non-starch polysaccharides, arabinoxylan, and β-glucan, which can form highly viscous solutions, reducing the leaching rate of ME, filtration rate, and the finished beer quality (Jamar et al., 2011). So, low VIS in malt is necessary for high malting quality. Although β-glucan is a primary component in cell wall, it is not the only parameter controlling VIS. At present, a number of QTLs regulating VIS have been identified (Table 1). Laidò et al. (2009) mapped two major QTLs, loci *Qvis1.1* and *Qvis5.1*, which were located on 1H at 60.3 cM and 5H at 137.5 cM, and explained as high as 39.5% and 24.3% of the variances, respectively. von Korff et al. (2008) also detected a QTL on 1H at 68 cM closely to *Qvis1.1.* Kochevenko et al. (2018) positioned a QTL *QVIS-3* on 3H (63.66 cM) explaining 13.82% of the phenotypic variance, and another QTL with slight phenotypic variance was found at the top of 4H. Wang J. et al. (2015) discovered locus *QVi.NaTx-7H* at 20.79–27.87 cM on 7H, contributing to 14.5% of the phenotypic variance. In addition, locus *QVi.NaTx-1H*, contributing to 17.8% of the variance, was detected at 61.15 cM on 1H, which may be the same QTL as *Qvis1.1* (Wang J. et al., 2015).

### Wort β-Glucan (WBG)

β-Glucan is mainly distributed in the aleurone layer and endosperm cell wall, accounting for 75% of the endosperm cell wall composition (Jamar et al., 2011). In the process of barley malt production and beer brewing, the incomplete degradation of endosperm cell wall will cause excessive WBG, which would influence the expansion of hydrolase and protease into the malt cells and decrease the extract content in the wort (Bamforth, 2003; Li et al., 2010; Bamforth, 2017). Meanwhile, excessive residual β-glucan in the malt will lead to an increase of VIS, which is not conducive to the filtration of wort and beer, and results in reduced beer quality (Vis and Lorenz, 1998; Bamforth, 2003). Therefore, reducing the VIS and improving the filterability of beer is significant for breeders and brewers.

Although WBG is affected by both genotypic and environmental factors, the genetic background is more significant (Jamar et al., 2011), and multiple QTLs for WBG have been identified using genetic populations (Table 1). Panozzo et al. (2007) located a QTL on 1H at 59 cM, accounting for 36.0–52.8% of the variance. Cu et al. (2016) located a QTL on 1H at 50.56 cM that explained 13.92% of the variance. Mohammadi et al. (2015) also detected a QTL on 1H at 58 cM using six-row spring breeding lines, which resulted in high reduction of WBG. In fact, the genetic distances of the three QTLs are relatively close, suggesting that they may be the same QTL. In addition, a genetic locus affecting WBG was positioned on 2H at 65.6 cM, which overlapped with the QTL interval (Adh8-ABGOI9) controlling grain β-glucan content. It is speculated that these two loci constitute the same QTL and can control both wort and grain β-glucan content (Han et al., 1995; Panozzo et al., 2007). Seventy-seven lines were evaluated for malting quality and selected for WBG analysis, and a QTL was mapped on 5H at 187 cM that can significantly reduce WBG (Mohammadi et al., 2015). Using the DH population from the Japanese barley variety “Mikamo Golden” and the North American variety “Harrington,” Zhou et al. (2012) identified a QTL on 6H at 35–55 cM and explained 17.2% of the variance. Kim et al. (2011) detected two QTLs on 7H using F5-derived lines, and accounted for 44.4% and 31.6–37.6% of the variances, respectively. Several genes have been cloned in barley grains by comparative genomics method, such as the *CslF* gene cluster on 2H and *HvCslF6* on 7H, which are involved in the synthesis of β-glucan (Han et al., 1995; Burton et al., 2006; Taketa et al., 2011). Loss of function of *CslF* genes can decrease the β-glucan content significantly.

Malt β-glucanase plays a critical role in the degradation of β-glucan during malting process (McCleary and Shameer, 1987; Han et al., 1995). Two β-glucanase genes, *Glb 1* and *Glb 2*, were cloned from barley malt and located on 5H and 1H, explaining 12.3% and 10.8% of the variances, respectively (Han et al., 1995; GrainGenes 3.0^2^). In addition, Han et al. (1995) detected a QTL on 4H at 79.5 cM that explained 12.6% of the variance. Panozzo et al. (2007) detected two major QTLs, one was detected on 1H at 50.9 cM, and the other was mapped on 4H at 22.9 cM. The major QTLs on 4H were detected under a variety of conditions, which indicated that the effect of environment on these QTLs was relatively low.

### Free Amino Nitrogen (FAN)

Free amino nitrogen is the only nitrogen source for yeast cell growth and reproduction, and its content in the wort plays a decisive role in yeast growth, synthesis, and metabolite changes (Stewart et al., 2013). FAN not only provides nutrition for yeast during beer fermentation, but also constitutes the flavor substance of beer. Thus, FAN is a significant indicator of beer quality. Although high PC in the grains can increase the FAN content, it also leads to a decrease of ME (Qi et al., 2005). Therefore, FAN in the wort is generally maintained at 180–220 mg/L.

In the genetic analysis of FAN (Table 1), Panozzo et al. (2007) detected four major QTLs, of which two were from the same DH lines linked to the Bmag0211 and Bmag0345 loci on 1H, which contributed to as high as 64.0% and 39–60% of the phenotypic variances, respectively. In another DH population, Panozzo et al. (2007) also identified two loci on 3H and 5H, with a lower variance compared with the first DH population. Cu et al. (2016) mapped two major QTLs, and one was located at 50.56 cM on 1H, near the Bmag0211 interval, another one was narrowed to an interval of 66.16–73.26 cM on 7H (Cu et al., 2016). Islamovic et al. (2014) positioned a major QTL on 7H at 34 cM, which was overlapped with QAn.NaTx-7H, a QTL located by Wang J. et al. (2015). The QTL on this location explained about 15% of the phenotypic variance at two different isolation populations.

### Soluble Protein (SP)

Soluble protein content in the wort is a parameter for evaluating wort quality. It affects the nutritional composition, flavors, foam, and abiotic stability of beer in the brewing process.

A number of QTLs for SP have been identified using different genetic populations, and the QTLs with higher contribution rates were detected on 1, 3, 5, and 7H (Table 1). Matthies et al. (2014) analyzed 174 European barley cultivars by GWAS and identified two QTLs, located on 1H at 94.9 cM, and 5H at 184.4 cM, respectively. A QTL was detected on 1H at 50.56 cM under different environmental conditions, and the QTL linkage marker 009-148 was not only linked to the traits controlling SP, but also related to FAN and KI. It is speculated that this region should be a critical site for malting quality (Cu et al., 2016). In addition, three major QTLs, contributing to 27.31%, 33.6%, and 29% of the phenotypic variances, were identified on 3H, 5H, and 7H, respectively (Mather et al., 1997; Zhou et al., 2016; Kochevenko et al., 2018). These reported QTLs contribute high variances and can be used for marker-assisted selection (MAS) improving the SP content of the malt.

### Protein Content (PC)

Protein is one of the main components of malt products. In the malting process, excessive GP will reduce ME, increase wort VIS, and decrease beer stability. At the same time, proteolysis can provide the only nitrogen source for yeast growth and various hydrolytic enzymes for starch degradation, and thus the PC of high-quality barley is generally 9–12% (Mather et al., 1997).

Protein content is extremely susceptible to environmental factors, and up to now, only a few QTLs with high contribution rates for malt protein content (MPC) and grain protein content (GPC) have been reported (Mather et al., 1997) (Table 1). Using a DH population, only *qPC2.1* and *qPC7.1*, associated with MPC, were identified on 2H and 7H, and explained 14.72% and 12.02% of the variances, respectively (Li et al., 2005). Although there is no significant difference between the total protein content in the barley grains and in the malt, the proportion of protein components is altered following germination (Celus et al., 2006), and more QTLs for GPC are detected (Table 1). Laidò et al. (2009) located two QTLs, *qPC1.1* and *qPC6.1*, on 1H and 6H that explained 29.1% and 18.7% of the variance, respectively. Using two DH populations, Elía et al. (2010) mapped seven QTLs on 1, 2, 3, 5, and 7H, explaining 13.4% to 62.3% of the variance. However, only three were identified in more than two environmental conditions, and a QTL at 82 cM on 2H was detected in all the planting conditions, explaining an average of 54% of the variance (Elía et al., 2010).

## Correlation Analysis of Malting Quality Traits in Barley

**FIGURE 1:**
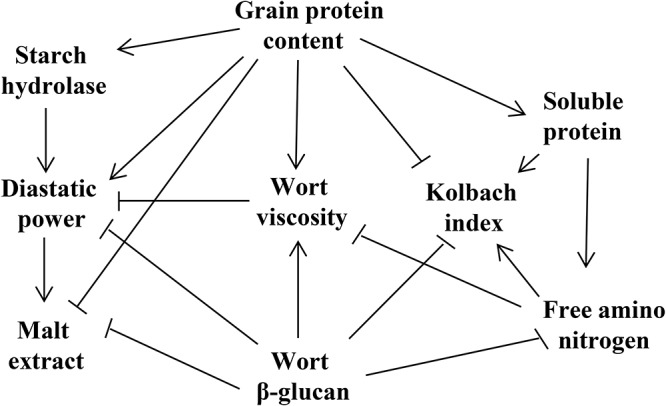
The correlation of malting quality traits. Arrows indicate positive interactions between parameters, and terminally barred lines indicate negative interactions.

The genetic elements controlling malting quality traits interact with each other to form a regulatory network that determines the malting quality (Figure 1). Among them, GPC has a great influence on the hydrolase activity, malt saccharification, beer fermentation and the biostability of the finished beer (Bond et al., 2015). In addition, GPC is also an essential factor regulating the leaching rate of ME. These two indicators are negatively correlated, and a high GPC can lead to reduced ME in the malt (Qi et al., 2005; Mohammadi et al., 2015). During the hydrolysis of malt starch, GPC can provide an abundance of enzymes for starch degradation, including α-amylase and β-amylase (Mather et al., 1997; Elía et al., 2010), and thus GPC is positively correlated with DP, but negatively correlated with KI. During germination, proteins in the grains are decomposed into amino acids by protease, which can provide a nitrogen source for the growth of beer yeast, but the correlation between GPC and FAN is low (Stewart et al., 2013; Pauli et al., 2015; Cu et al., 2016).

Soluble protein, derived from GP, produces various amino acids and small molecular peptides following degradation. Therefore, SP is significantly positively interacted with GPC, FAN, and KI (Cu et al., 2016). FAN is mainly used in the subsequent beer fermentation process. Although FAN is negatively interacted with VIS and WBG, there is no direct relation with the two traits. Thus, it is supposed that FAN may indirectly influence VIS and WBG by altering the PC. KI was found to be negatively interacted with GPC and VIS, and positively correlated with SP and FAN with a high correlation coefficient (Wang J. et al., 2015; Cu et al., 2016).

Wort β-glucan is negatively interacted with most malting quality traits (Pauli et al., 2015). Excessive WBG is the residue of the incomplete degradation of cell wall, which may affect the expansion of various hydrolases in the germinated grains and reduce the ME content. Additionally, WBG is also significantly negatively correlated with FAN, KI, and SP, and positively interacted with GPC (Pauli et al., 2015). High-quality malt requires lower wort VIS, and excessive residues of β-glucan will increase wort VIS and lead to filtration issues (Bamforth, 2003). Thus, WBG is significantly positively correlated with VIS, both of which are unfavorable traits for improving malting quality. In addition, a high VIS will result in increased beer turbidity (Wei et al., 2009).

Diastatic power, representation of the starch hydrolase activity, is positively correlated with starch hydrolases (Gibson et al., 1995; Mohammadi et al., 2015). In addition, DP is positively interacted with GPC, FAN, and ME, but significantly negatively correlated with WBG and VIS (Emebiri et al., 2004; Pauli et al., 2015).

## Improving Malting Quality in Barley and Research Prospects

### Selection for Improved Traits

Malting quality in barley is a comprehensive reflection of many indicators, which are easily influenced by environmental conditions. However, there are also differences among varieties. For example, Molina-Cano et al. (1997) detected ME, wort VIS, KI, and DP in numerous varieties and found that there were highly significant differences among the four quality traits, of which VIS and ME were particularly correlated with the genotype. High-quality malt requires high ME and DP, optimal PC, and low WBG. However, it is difficult to improve multiple traits simultaneously in breeding because of a longer breeding cycles, so improving single or several traits is necessary during the improvement process.

**FIGURE 2:**
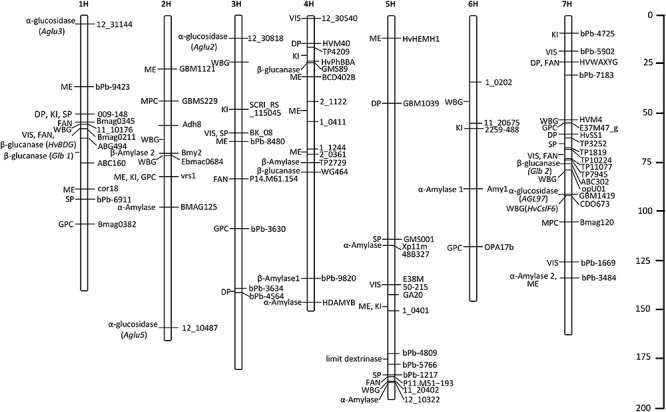
The distributions of QTLs/genes accounting for high phenotypic variances. ME, malt extract; DP, diastatic power; VIS, wort viscosity; KI, kolbach index; FAN, free amino nitrogen; SP, soluble protein; WBG, wort β-glucan; MPC, malt protein content; GPC, grain protein content.

In this review, we listed the QTLs/genes identified in recent years and located them on seven chromosomes (Table 1 and Figure 2), and found that most QTLs/genes controlling malting quality were mainly positioned in the intervals of 1H, 4H, 5H, and 7H, which indicates that malting quality traits with high phenotypic variances may prefer to cluster in these regions. Though improvement of a few traits was considered as the first choice in the past, substitution of a large interval containing elite traits in barley will be also feasible in the future, which can achieve the breeding programs in short term.

Previous studies have provided valuable experience for traits improvement. ME mainly influenced by the genetic background, is the most significant element determining beer yield in the malting process, thus it is a quantitative trait that is extremely easy to improve (Sarkar et al., 2008). DP is also proportional to the yield and quality of beer brewing (Henson and Duke, 2007), and the correlation coefficient between DP and the three main amylases, α-amylase, β-amylase, and limit dextrinase, was as high as 0.79 (Gibson et al., 1995). Among these, β-amylase is the most important enzyme affecting DP, followed by α-amylase (Clancy et al., 2003; Henson and Duke, 2007). Therefore, the applications of these two hydrolases can significantly improve the DP level. During malting, GPC is not only altered by its own genetic basis, but also more susceptible to environment. It is necessary to coordinate the relationship between GPC and malting quality through genetic improvement or cultivation (Mather et al., 1997). WBG is negatively correlated with most barley quality traits, and reducing its content is also favorable. The cultivation of barley varieties with low BG content or high β-glucanase activity will facilitate to reduce the β-glucan content in the wort.

In our review, more than 60 QTLs/genes were listed, in which 15 genes have been cloned, and others were located in intervals between genetic markers. In molecular breeding, genes with high heritability will be better than identified QTLs because selection targets are more accurate and clear. Secondly, QTLs explaining high variances will also be chosen for high-quality breeding, because these QTLs will not be seriously influenced by the environment or other traits. Therefore, DP and WBG must be better choices for the improvement of malting quality, then VIS should be considered according to Table 1. In addition, the proportion of malting quality parameters in barley breeding is different, due to the standards set by different countries or regions. Breeders and scientists in different countries should adjust their strategies to improve malting quality according to their own national industrial standards and purposes (European Brewery Convention, 1998; Laidò et al., 2009; Punda, 2009).

### MAS for Malting Quality Breeding

The corresponding phenotypes of malting quality traits in barley are affected by both genetic and environmental elements, and positive and negative interactions among these traits are also present, which increase the difficulty for breeding application. Barley, a diploid cereal crop, has a large genome size of 5.1 Gb and highly repetitive DNA composition (Mascher et al., 2017), which also adds interference from multiple micro-effective homologous genes. The use of conventional breeding techniques to improve malting quality will face a series of difficulties, such as low selection efficiency and longer selection cycles, and it is difficult to aggregate multiple quantitative trait genes. With the discovery of regulatory mechanisms of major malting quality traits, combining MAS with conventional techniques to construct economical and efficient molecular breeding technology systems has become an important research direction in malt barley breeding.

Marker-assisted selection is convenient for breeding of malting barley and have been applied successfully. For example, Xu et al. (2018) transferred a thermostable β-amylase from wild barley into a commercial variety, and identified several elite lines with high DP. In the barley MAS procedures, elite barley varieties without serious defects and donor parent containing favorable alleles should be selected for the improvement of malting quality. Then, polymorphic markers closely linked to the alleles needs to be designed for the identification of hybrid plants. Hybridization and backcross must be carried out for transferring alleles from donor parents to elite varieties, and MAS will be implemented for selection of superior alleles in the segregating population.

In this review, we analyze the main malting quality indicators, ME, DP, VIS, KOL, FAN, GPC, SP, and WBG, and their correlation with each other. In addition, we also find that starch hydrolase, β-glucan, and PC have greatest impact on malting quality and play important roles in the regulatory network. Although the target traits can be selected quickly by MAS, but it also may be difficult to improve the traits controlled by QTLs with a low genetic contribution. Meanwhile, these traits are easily affected by environmental factors, which may weaken the improvement effect. Here, we list the major QTLs/genes that regulate the eight indicators (Table 1 and Figure 2), particularly those major loci that contribute to large phenotypic variances and have been detected in multiple populations or environmental conditions, which may provide more genetic information to breeders for facilitating the targeted improvement of malting quality.

## Author Contributions

DX and YF conceived the work and wrote the manuscript. YF and XZ prepared the figures and tables.

## Conflict of Interest Statement

The authors declare that the research was conducted in the absence of any commercial or financial relationships that could be construed as a potential conflict of interest.

## Footnotes

1 http://faostat.fao.org

2 https://wheat.pw.usda.gov/GG3/
